# The Quest for Type 2 Diabetes Subgroups Identification: Literature Review for a New Subtype Proposal

**DOI:** 10.7759/cureus.3770

**Published:** 2018-12-24

**Authors:** Mohamed Islam Delma

**Affiliations:** 1 Internal Medicine, Colonel Chaabani Hospital, El Menia, DZA

**Keywords:** type 2 diabetes, alpha 1 adrenoreceptor, silent myocardial infarction, diabetic nephropathy, sympathetic innervation, diabetes complications, diabetic neuropathy

## Abstract

Type 2 diabetes is considered typically as a heterogeneous disease that englobes the potential different subtypes with distinct pathophysiological mechanisms and/or susceptibility to complications. Some authors have succeeded in the identification of some of these subgroups, but a lot of work remains to be completed. Given the effects of the sympathetic innervation via alpha 1 adrenoceptors on diabetes target organs and the interindividual variability of this receptor sensitivity, the existence of a subtype of type 2 diabetes with hyperactivation of alpha 1 adrenoceptors (HA1A) is proposed. Based on the literature review, the potential characteristics of this phenotype and its susceptibility to certain complications have been identified in this article.

## Introduction and background

Diabetes mellitus is a metabolic disorder characterized by hyperglycemia. The diagnosis is established based on well-defined biologic criteria. Type 2 accounts for 90% to 95% of all diabetes and is caused by insulin deficiency associated with insulin resistance [1]. It has become clear that type 2 diabetes is a heterogeneous disease englobing different groups with distinct physiopathology, evolution, and susceptibility to complications [2-3]. These subgroups may be defined by either a common pathophysiological mechanism or susceptibility to specific complications. Identifying these groups seems to be the gateway for a physiopathology-based therapeutic approach and complication-oriented screening for timely and better management. In fact, much effort has been made in this direction. Li and his colleagues have distinguished three subtypes associated with a distinct type of complications [3], while the study carried by Ahlqvist et al. identified five clusters of patients with a different pattern of complications [2].

Long ago, the pathogenic role of the sympathetic innervation via adrenoceptors in hyperglycemia had been established [4-5]. Of note, an interindividual variability of alpha 1 adrenoceptor subtype activity had also been reported [6]. 

Based on this background, this study developed a hypothesis of the existence of a type 2 diabetes subtype with hyperactivation of the alpha 1 adrenoceptors (HA1A).

## Review

Overview of alpha 1 adrenoceptors

Generality

Based on the original research studies, Calzada et al. have provided a systematic review of the adrenoceptor subtypes [7]. In summary, adrenoceptors are membrane receptors responsible for mediating responses to the endogenous catecholamines including noradrenaline and adrenaline. Initially, they were classified as alpha receptors with preponderant excitatory effects and beta receptors with marked inhibitory effects. Subsequently, subtypes for alpha and beta receptors were discovered. Alpha receptors were subdivided into alpha 1 and alpha 2, based on their pharmacologic proprieties. Subsequently, with the advancement of pharmacologic and cloning techniques, subdivisions of alpha 1 and alpha 2 were established. Alpha 1 adrenoceptors are subdivided into alpha 1a, alpha 1b, and alpha 1d subtypes, but their distinct distributions and effects are not fully understood. Hence, in this article, they have been referred to as alpha 1 adrenoceptors.

Alpha 1 Adrenoceptor Activity Evaluation

A method for the evaluation of alpha 1 adrenoceptor activity is via the assessment of human dorsal hand vein alpha-receptor activity [6]. The amplitude of dorsal hand vein vasoconstriction induced by alpha 1 agonist phenylephrine is measured by a device called the linear variable differential transformer. The dose of the alpha 1 agonist that induces 50% of vasoconstriction is used to compare individual sensitivity of alpha 1 adrenoceptors [6].

Another method consists of measuring the dose of phenylephrine required to elevate the mean arterial pressure by 25 mmHg [8].

The results of these techniques have confirmed the interindividual variability of alpha 1 adrenoceptor sensitivity [6,9]. In the next section, the characteristics of type 2 patients with HA1A have been proposed.

Characteristics of type 2 diabetes with alpha 1 receptor hyperactivation

Demographic Parameters

Although some authors have found that some parameters are associated with alpha 1 adrenoceptor sensitivity such as sex and ethnicity, these findings were not consistent among all studies [9].

Anxiety

If we refer to the role of sympathetic innervation on anxiety and the experimental findings of the involvement of the alpha 1 adrenoceptors in stress-induced anxiety-like responses [10-11], we may expect that anxiety would be a trait of patients with this subtype.

Diabetogenic Effect

If the two mechanisms unanimously incriminated in type 2 diabetes and the defects in insulin secretion and exaggerated insulin resistance are considered alone, alpha 1 adrenoceptor hyperactivation would have only a mild diabetogenic effect, as its effect on these two mechanisms is only moderate and/or inconsistent among studies [12-13]. But if the role of glucagon on type 2 diabetes is taken into account [14], the effect of HA1A would be important, as this receptor induces glucagon secretion and potentiates the activity of adenylyl cyclase, a mediator of the glucagon receptor signaling pathway [15-16]. Overall, the development of type 2 diabetes in individuals with HA1A would be conditioned by the presence of an additional predisposing factor.

Due to the sympathetic system activation in the postprandial period [17], the characteristic postprandial glycemic profile in patients with HA1A subtype would be an accentuation of the postprandial hyperglycemia, followed by a relative decrease in the glycemic value. The first effect would be due to the potentiation of postprandial glucagon-induced hyperglycemia, while the second would be caused by the increase in glucagon effect on insulin secretion and is dependent on insulin reserve [18-19]. Furthermore, this profile may be present even in prediabetes, as it would be attested in glucose tolerance test (HGPO).

Lipidic Profile

Pool and his colleagues have demonstrated that alpha 1 receptor blockade induces a rise in high-density lipoprotein (HDL) levels and a reduction in low-density lipoprotein (LDL) levels and apolipoprotein B (apo B) [20]. This suggests that alpha 1 adrenoceptor stimulation may exercise the opposite effect. The patients with type 2 diabetes with HA1A are expected to have a thrombogenic lipidic profile with low HDL levels and high LDL and apo B levels. If this assumption is confirmed, the outcomes of alpha 1 adrenoceptor blockade in this subgroup, especially in patients with the highest cardiovascular risk, should be evaluated.

Concerning triglyceridemia, patients with HA1A subtype may have a tendency to hypertriglyceridemia. This is expected due to the hepatic alpha 1 adrenoceptor role in triglyceride liberation from the liver [21-22]. 

Silent Myocardial Infarction

Silent myocardial infarction (SMI) is a myocardial ischemic event without pain. Systematic screening for this condition by stress tests in diabetic patients is subject to debate [23]. Some authors advocate the screening program, given the high prevalence of cardiovascular diseases and SMI in patients with diabetes and the possible complications of undetected myocardial ischemia. Others argue against it, as there is not yet clear evidence of benefits from this systematic screening and consequent invasive therapeutics compared to good medical control of cardiovascular risk factors. But, how about patients with HA1A? Given the opposition of alpha 2 receptor analgesic effect by alpha 1 receptor [24] and the role of the former on SMI [25], myocardial infarction is more likely to be symptomatic than asymptomatic in diabetic patients with HA1A. If confirmed, this argument would advocate against systematic screening for SMI in HA1A subtype.

Erectile Dysfunction

The effect of sympathetic innervation via alpha 1 adrenergic receptors on penis detumescence through corpus cavernosum contraction is well-known [26]. This is the rationale of alpha 1 receptor blocker use in erectile dysfunction [26-27]. It is expected that patients with HA1A subtype would be more prone to this diabetes complication. A trial of the effect alpha 1 blocker on these patients should be evaluated via clinical trials.

Painful Neuropathy

The role of sympathetic innervation in this complication was suggested by the findings of a higher level of plasma norepinephrine in painful than in painless diabetic neuropathy [28]. The role of alpha 1 adrenoceptors seems to be primordial since it has been shown that nerve damage induces the upregulation of alpha 1 adrenoceptors that are responsible for exaggerating the adrenergic component of pain [29]. Patients with HA1A subtype are more likely to express nerve damage caused by chronic hyperglycemia as painful than painless neuropathy.

Another diabetes-related neuropathy is treatment-induced neuropathy, also known as insulin neuritis. It is defined as a painful, autonomic neuropathy that develops in the setting of rapid improvements in glycemic control in individuals with a long history of hyperglycemia [30]. The physiopathology is not completely clear, and some authors suggest a neural damage mechanism [31]. If this suggestion is validated, we may expect a susceptibility of HA1A subtype to this treatment-induced neuropathy.

Diabetic Retinopathy

Diabetic retinopathy is a microvascular complication of diabetes. Du et al. had shown, in their experimental study, that the alpha 1 adrenoceptor blockers inhibit the important features of diabetic retinopathy such as superoxide generation, proinflammatory protein expression, and the degeneration of retinal capillaries [32]. If their results are replicated in clinical studies, we expect that the blockade of alpha 1 adrenoceptors will be more beneficial in diabetic patients with HA1A.

Diabetic Nephropathy

Alpha 1 adrenoceptor autoantibodies have been objectified in some patients with diabetic nephropathy, with deleterious effects on kidney and arterial pressure [33-34]. These autoantibodies seem to be of stimulatory nature, as their effects are contracted by the alpha 1 adrenoceptor antagonists [33-34]. This led to the suggestion that HA1A may be harmful to the kidney. Hence, the clinical outcomes of alpha 1 adrenoceptor blockade on diabetic nephropathy prevention or regression should be evaluated in these type 2 patients with HA1A.

In summary, this paper aimed to develop the hypothesis of the existence of type 2 diabetes with HA1A subgroup, characterized by a common diabetogenic mechanism via glucagon synergism, and more importantly, a specific pattern of complications. Eventually, patients with this subtype may need an additional therapeutic option consisting of alpha 1 adrenoceptor blockade. This hypothesis is based on the literature review of different alpha 1 receptor effects, notably on diabetes target organs. However, some limitations should be considered. First, the aforementioned tests of alpha 1 receptor sensitivity evaluation may be specific of only one subtype of these receptors [9]. Given the lack of dispensible tests for all alpha 1 receptor subtypes, it is postulated in this paper that hyperactivation of one of them may reflect hyperactivation of the whole family. Eventually, if specific tests are developed, this suggestion should be reevaluated. Secondly, the existence of additional diabetes risk factors for each patient with HA1A is suggested, but we have omitted the potential influence of these factors on the evolution pattern of this subtype. This is helpful initially for a better characterization of this subtype, but these factors impact should be discussed later in a case per case basis.

## Conclusions

Nowadays, type 2 diabetes is considered as a heterogeneous group encompassing different phenotypes. Identification of these phenotypes is the cornerstone of the personalized management of type 2 diabetes advocated by scientific societies. This paper aimed to identify the characteristics of a potential new subtype: type 2 diabetes with HA1A. Clinical studies are needed for confirmation or invalidation of this hypothesis.
